# Comparing the Ratio of Therapist Support to Internet Sessions in a Blended Therapy Delivered to Trauma-Exposed Veterans: Quasi-experimental Comparison Study

**DOI:** 10.2196/33080

**Published:** 2022-04-27

**Authors:** Marylene Cloitre, Amber Bush Amspoker, Terri L Fletcher, Julianna B Hogan, Christie Jackson, Adam Jacobs, Rayan Shammet, Sarah Speicher, Miryam Wassef, Jan Lindsay

**Affiliations:** 1 National Center for PTSD Dissemination and Training Division, Veterans Administration Palo Alto Health Care System Palo Alto, CA United States; 2 Veterans Administration Health Services Research & Development Center for Innovations in Quality, Effectiveness and Safety Michael E DeBakey Veterans Affairs Medical Center Baylor Medical College Houston, TX United States

**Keywords:** PTSD, depression, veterans, blended therapy, iCBT, web-based, webSTAIR, noninferiority, mental health, digital health

## Abstract

**Background:**

Blended models of therapy, which incorporate elements of both internet and face-to-face methods, have been shown to be effective, but therapists and patients have expressed concerns that fewer face-to-face therapy sessions than self-guided internet sessions may be associated with lower therapeutic alliance, lower program completion rates, and poorer outcomes.

**Objective:**

A multisite quasi-experimental comparison study with a noninferiority design implemented in routine clinical care was used to assess webSTAIR, a 10-module blended therapy derived from STAIR (skills training in affective and interpersonal regulation) for trauma-exposed individuals delivered with 10 weekly therapist sessions (termed Coach10) compared to 5 biweekly sessions (Coach5). It was hypothesized that Coach5 would be as good as Coach10 in a range of outcomes.

**Methods:**

A total of 202 veterans were enrolled in the study with 101 assigned to Coach5 and 101 to Coach10. Posttraumatic stress disorder (PTSD) symptoms, depression, emotion regulation, interpersonal problems, and social functioning measures were collected pre-, mid-, and posttreatment, and at a 3-month follow-up. Noninferiority analyses were conducted on symptom outcome measures. Comparisons were made of continuous and categorical measures regarding participant and therapist activities.

**Results:**

Participants reported moderate to severe levels of baseline PTSD, depression, or both. Significant reductions were obtained in all symptom measures posttreatment and at the 3-month follow up. Coach5 was not inferior to Coach10 in any outcome. Therapeutic alliance was at an equivalently high level across the 2 treatment conditions; completion rates and web usage were similar. Total session time was substantially less for the Coach5 therapists than the Coach10 therapists. Both programs were associated with a low, but equal number of therapist activities related to scheduling and crisis or motivational sessions.

**Conclusions:**

A blended model delivered with 5 sessions of therapist support was noninferior to 10 sessions in individuals with moderate to severe symptoms. Future studies identifying patient characteristics as moderators of outcomes with high versus low doses of therapist support will help create flexible, technology-based intervention programming.

## Introduction

### Background

Meta-analyses and reviews have found that trauma-exposed individuals receive moderate benefits for posttraumatic stress disorder (PTSD) and depression symptom reduction from internet-based interventions; larger effects were found in studies that included therapist support as compared to completely self-guided approaches [1-3]. Nevertheless, internet interventions, even those with some therapist support, do not provide full recovery for everyone [1,3], particularly over the long term [2], indicating the value of further exploration and improvement in technology-supported treatments. An emerging alternative approach is the “blended intervention,” which integrates face-to-face therapy with internet approaches. Blended therapy is characterized by continued therapist input alongside internet self-help to allow greater flexibility and personalization within the overall therapy process [4,5]. Blended therapies provide an alternative model of care which may improve treatment outcomes as well as increase engagement with mental health services among those who prefer more intensive therapist guidance.

Blended therapy approaches have been found to be positively viewed by both therapists and patients and preferred over stand-alone internet programs [4-7]. To date, several case reports and open trials of individuals with anxiety or depression have reported that blended interventions that focus on web-based interventions but also provide substantial therapist support are feasible and highly acceptable to clients [8-11]. Three randomized controlled trials (RCTs) have supported their efficacy. An early study comparing subjects who received an intervention to those who were placed on a waitlist found moderate to large between-group effect sizes for clients with social phobia who received 9 weeks of internet-based cognitive behavioral therapy (CBT) integrated with two 3-hour exposure sessions and email support from a therapist [12]. An RCT of problem-solving therapy (PST) for individuals with anxiety or depression found that receiving web-based PST with support was superior to being placed on a waitlist, while 3 other forms of delivery—web alone, with support as requested, or with weekly emails—were not [13]. Last, a 4-arm RCT, which included patients with anxiety or depression, found that the blended approach was superior not only to the no-treatment condition but also to a face-to-face–only condition and internet-only condition [14]. The findings from the latter two studies suggest the possibility of synergistic effects in combining these two intervention approaches.

Based on the above successes, investigation of blended therapies for trauma-exposed populations is justified. A recent open trial study assessed patient satisfaction and outcomes in a blended model delivered to rural trauma-exposed veterans. The program was entirely virtual; the patients completed the web-based program concurrent with a face-to-face coaching session via video conferencing [15]. The program, webSTAIR, is a 10-session, transdiagnostic, trauma-informed program derived from STAIR (skills training in affective and interpersonal regulation), a CBT approach with empirical support [16]. Significant improvements in PTSD, depression, and social functioning were obtained with moderate to large effect sizes at posttreatment and at a 3-month follow up. Analyses of posttreatment interviews revealed themes regarding the value and importance of the therapists, particularly regarding their ability to provide support, accountability, and effective tailoring of the interventions, activities that have been described as key therapist functions in technology-based interventions [17].

### Objectives

One important long-term goal is to identify the optimal amount of therapist support relative to self-guided work to maximize outcomes. Both patients and therapists tend to take a “more is better” perspective when considering the presence of therapists in blended treatments. Results from a Delphi survey study found that therapists preferred that 75% of sessions be face-to-face, while most patients preferred 50% to 60% [18]. Moreover, both patients and therapists express concern that less therapist involvement will be associated with lower therapeutic alliance, lower completion rates, and reduced effectiveness [19,20]. To our knowledge, however, no studies have been conducted that consider the impact of the amount of therapist support (ie, number of sessions) on outcomes.

The purpose of this study was to systematically assess and compare the impact of 2 different ratios of therapist sessions to self-guided work on therapeutic alliance, completion rates, and symptom reduction among trauma-exposed veterans. The specific goals of the project were (1) to replicate the results of the first webSTAIR study by using a 1:1 ratio of therapist sessions to self-guided web-based sessions and (2) to assess outcomes where therapist support was reduced by 50%, (ie, the ratio was 1:2), with 1 therapist session for every 2 self-guided web-based sessions. The 1:1 ratio was adopted as the anchoring reference point for this investigation based on the high patient satisfaction ratings and the large effect sizes reported in the first webSTAIR study, which used this ratio [15], and because this ratio provided the maximum number of therapist sessions in a blended therapy program in which the intervention of interest was the web-based intervention. The selection of the 1:2 ratio was based on a pilot study that delivered webSTAIR with this ratio, yielding significant symptom reduction with a large effect size [21]. That study also included posttreatment interviews that identified an association between patient satisfaction and the number of therapist sessions; veterans reported in the interviews that self-guided work fostered independence and mastery.

We hypothesized that providing 1 therapist session for every 2 self-guided sessions in this 10-module treatment (ie, the webSTAIR Coach5 condition) would not be inferior to providing 1 therapist session for each self-guided session (ie, the webSTAIR Coach10 condition) in regard to PTSD, depression, emotion regulation, interpersonal problems, or social functioning outcomes. We also compared therapeutic alliance, completion rates, and web usage (measured as time in minutes) among the participants. The amount of time therapists spent in session and the amount of activity related to rescheduling appointments and providing additional sessions as needed for crisis management and motivational support was also compared.

## Methods

### Study Design

The study was funded by the Office of Rural Health and dedicated to rural veterans, with a focus on rural women veterans, who have been identified as under-represented in mental health services relative to both urban male and female veterans [22]. This was a naturalistic evaluation study, in which delivery of the program was conducted as part of routine care in mental health outpatient clinics within the Veterans Health Administration. The study used a quasi-experimental comparison design, in which 9 service sites were assigned to either the Coach5 or the Coach10 condition, matched on 3 characteristics: percentage of rural veterans enrolled in the mental health service, projected number of veterans expected to be enrolled in the study per month, and job description of the therapists trained to deliver the intervention (eg, psychologist, social worker, or mental health technician).

### Procedures

Candidates for the program were referred by clinical therapists. Individuals were eligible for inclusion if they reported a history of trauma exposure and were currently experiencing symptoms of PTSD, depression, or both, as indicated by a positive screen on the Primary Care PTSD Screen [23] or the 2-item Patient Health Questionnaire (PHQ-2) [24]. Additional inclusion criteria were an expressed willingness to complete assessment and treatment procedures and an interest in working on improving emotion regulation skills and interpersonal relationships. Exclusion criteria were active suicidal or homicidal ideation, psychosis, mania, cognitive impairment, inability to attend regular telemental health appointments, primary substance or alcohol use difficulties, current interpersonal violence, lack of a private place to connect for sessions, engagement in concurrent trauma-focused treatment for PTSD, and receipt of inpatient or residential PTSD care in the past year. Although the target population for enrollment into the study was rural male veterans and rural women veterans, any veteran who satisfied the above criteria and could not easily access in-clinic care (because of, for example, health concerns, time constraints, or elder or childcare responsibilities) was accepted into the program.

### Outcomes

#### Assessment and Symptom Measures

All assessments were conducted by the study coordinator via telephone at pretreatment, the midpoint (session 5), posttreatment, and at the 3-month follow up. The initial assessment included an inquiry about frequency of traumatic events using an adapted version of the Diagnostic and Statistical Manual of Mental Disorders, Fifth Edition (DSM-5)-derived Life Events Checklist (LEC-5) [25]. PTSD symptoms were measured using the PTSD Checklist for DSM-5 (PCL-5) [26], depression was measured with the Patient Health Questionnaire (PHQ)-9 [27], emotion regulation with the Difficulties in Emotion Regulation Scale (DERS)-36 [28], interpersonal problems with the Inventory of Interpersonal Problems (IIP)-32 [29], and social functioning by the brief, 21-item version of the World Health Organisation Disability Assessment Schedule (WHODAS)-2 [15,30], designated as the WHODAS-21. Probable diagnoses of PTSD (PCL-5 score>33) and depressive disorder (PHQ score>10) were calculated at baseline. Therapeutic alliance was measured with the patient version of the Working Alliance Inventory (WAI) [31].

#### Participant Web Usage and Therapist Time and Activities

Usage data, such as total number of minutes each user engaged in webSTAIR, was a built-in analytic feature of the program. The therapists used an online survey not integrated with the webSTAIR site to log the number of minutes they were engaged with the veterans after each session as well as the number of times they engaged in additional interaction with the veteran since their last session. Interactions included phone calls, instant messages, and emails to reschedule appointments as well as crisis or motivational sessions deemed clinically relevant by the therapist. The therapists were allowed to spend up to 20 minutes for crisis or motivational sessions.

### WebSTAIR Intervention

The webSTAIR program consists of 10 web-based modules adapted from STAIR [32]. The first 5 modules review emotional awareness, emotion management, and distress tolerance while the final 5 modules raise awareness about relationship patterns and provide interpersonal skills training regarding effective assertiveness, interpersonal flexibility, and compassion for the self and others. Modules include text, video, and audio delivery of psychoeducation, as well as interactive exercises and worksheets to aid the patient in learning and practicing the material.

### Therapist Sessions and Adherence

The therapists followed a manual that provided instructions for each session. The sessions were 45-50 minutes long and were organized with the same general tasks and goals. The therapists had 4 tasks: to clarify key concepts presented in the modules, to reinforce engagement and enthusiasm for the material, to help the participant tailor the skills to their own life experiences and concerns, and to support the participant in completing the modules on a weekly basis. The overall goal of the sessions was to help the participants make the most of the webSTAIR material by reinforcing work they did independently. The Coach5 condition required that the therapists review materials from 2 modules in a session while the Coach10 condition involved review of materials from only 1 module. Topics and content covered by the instruction manuals did not differ between the 2 treatment conditions. The therapists completed self-reported adherence ratings after each session. Self-reported adherence ratings have been found to be reliable and were chosen as being appropriate for this resource-limited intervention approach [33].

### Therapists

All webSTAIR therapists were licensed mental health staff working in Veterans Health Administration clinics. Five were licensed clinical psychologists, 3 were licensed social workers, and 1 was a licensed professional mental health counselor. All the therapists were women. Training for Coach5 and Coach10 was implemented separately, and each group was provided their own manual describing the topics and content for each of their sessions. Training also covered unique issues related to the use of web-based technology in comparison to traditional face-to-face psychotherapy. The Coach5 and Coach10 therapists each received weekly group phone supervision sessions with an experienced clinical psychologist and certified STAIR trainer. The therapists also attended weekly implementation meetings to address questions and concerns related to implementing a web-based telemental health intervention.

### Statistical Analysis Plan

Study noncompleters were defined as those who did not complete either the posttreatment or 3-month follow-up assessments. Pretreatment demographic variables and clinical characteristics were compared in the 2 treatment groups (Coach5 and Coach10) with independent samples and a 2-tailed *t* test for continuous variables and the chi-square test for categorical variables. The pretreatment demographic variables and clinical characteristics of study completers and noncompleters were compared with similar analyses.

We then examined linear changes in outcomes over time separately within each treatment group. For the Coach5 and Coach10 groups, an unconditional linear growth curve model using Proc Mixed in SAS (version 9.4; SAS Institute, Inc) was employed to examine linear changes over time for each of the 5 outcomes. In each model, time was the sole predictor and was coded such that 0 equaled baseline, 1 equaled the midpoint assessment, 2 equaled the posttreatment assessment, and 3 equaled the 3-month follow-up assessment. Both the intercept and time were included as random effects in all models. For each condition, within-group effect sizes from pre- to posttreatment and from pretreatment to the 3-month follow-up were calculated. Unlike superiority trials, where an intent-to-treat (ITT) analysis is more conservative as it makes 2 groups more similar, for a noninferiority trial, an observed or per-protocol analysis yields a more conservative estimate as it exaggerates the differences between treatment groups [33]. Therefore, a series of ANCOVA models was first calculated using the observed data to examine the treatment conditions (ie, Coach5 and Coach10) as predictors of each outcome at the posttreatment assessment. Treatment-group comparisons of posttreatment assessment outcomes were then repeated with another set of ITT analyses that used the multiple imputation procedures Proc MI and Mianalyze in SAS. Respective baseline scores and characteristics that differed between the treatment conditions were included as covariates in all models. Similar observed and ITT analyses were then used to examine treatment group differences in the 3-month follow-up assessment.

Examination of between-group changes relative to noninferiority margins was accomplished with 4 steps. First, noninferiority margins (*F*) for the posttreatment and follow-up assessments were calculated for each outcome using data from the initial webSTAIR study [19]. For each outcome, we calculated the noninferiority margin, which was 80% of the change in Coach10 from baseline to the posttreatment assessment, and then calculated the margin from baseline to the 3-month follow-up assessment. Second, we examined the actual difference between Coach5 and Coach10 in the change from baseline to posttreatment and from baseline to the 3-month follow up using the differences in differences (ie, the change score) approach. Baseline was always coded 0, and the follow-up assessment (posttreatment or 3-month follow-up) was coded 1. For each of the 5 outcomes, Proc Mixed in SAS was employed with time, treatment group, and the time by treatment group interaction as predictors. The intercept was included as a random effect. A significant time by treatment group interaction indicated a significant difference between groups in the change in outcome. Of primary interest was the point estimate of the difference in differences. Third, we calculated the 95% CI for each difference in differences. Finally, we plotted the posttreatment estimate of the difference in differences and its 95% CI relative to the noninferiority margin.

If we consider the difference in effect between Coach5 and Coach10, superiority of Coach5 versus Coach10 implies that the lower bound of the 95% CI is greater than 0. Noninferiority of Coach5 allows the lower bound of the 95% CI to extend below 0, so long as it remains above the noninferiority margin and so long as the upper bound of the 95% CI is greater than 0. In the case that the lower bound of the 95% CI falls below and the upper bound of the 95% CI falls above the noninferiority margin, the study result is indeterminate. Finally, inferiority of Coach5 versus Coach10 is implied if the upper bound of the 95% CI falls below the noninferiority margin [34]. All analyses involved 2-sided significance testing and were conducted in SAS. Effect sizes were also calculated.

A series of 2-tailed independent sample *t* tests were conducted to examine mean differences between Coach5 and Coach10 in (1) participant therapeutic alliance score, (2) number of modules completed by participants, (3) average amount of time spent (in minutes) per module and in total across all modules, (4) total therapist time spent (in minutes) providing treatment, (5) number of times the therapist made a phone call, texted, or emailed to reschedule an appointment, and (6) the number of times the therapist provided an additional session to address a crisis or enhance motivation. The chi-square test was used to examine the difference between Coach5 and Coach10 in program completion rate.

### Ethics Approval

This project was funded by the Department of Veterans Affairs Office of Rural Health. All procedures involved in the evaluation were reviewed and exempted by the local academic institutional review board (APP03H08).

## Results

### Participants

**Figure 1:**
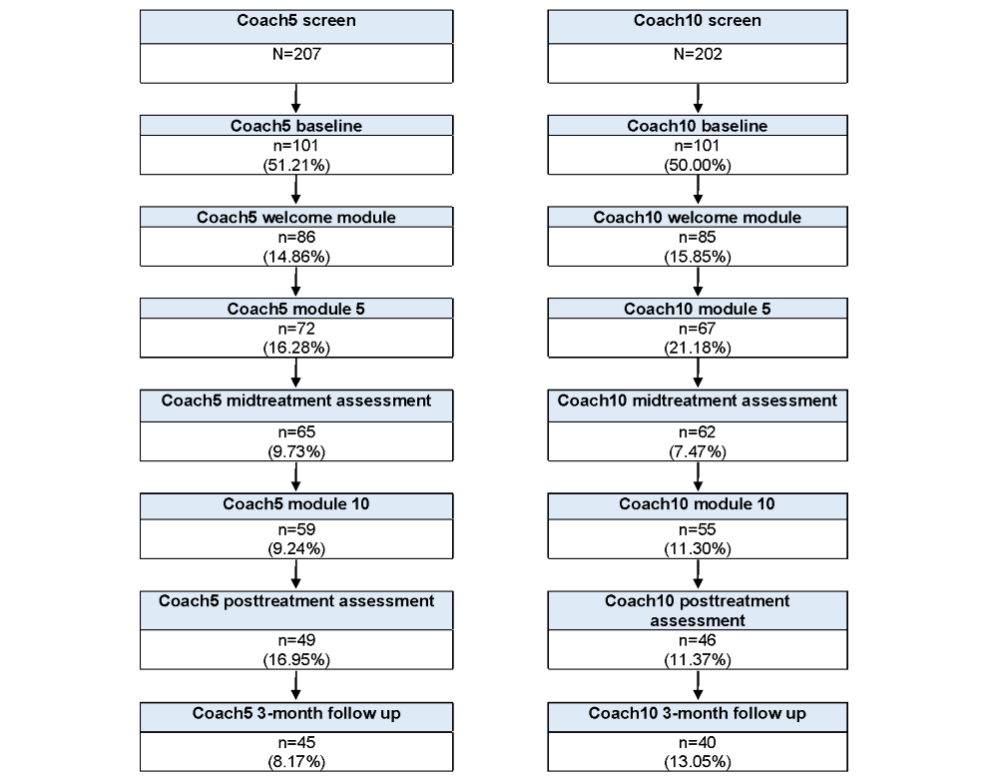
CONSORT chart for Coach5 and Coach10 conditions.

Given the quasi-experimental design, we wished to assess the similarity of the 2 conditions in enrollment rates and participant characteristics. As indicated by the Consolidated Standards of Reporting Trials (CONSORT) chart (Figure 1), there were no differences between the 2 treatment conditions in enrollment numbers, attrition rate from screening to baseline assessment, or from baseline assessment to program enrollment. The percentages in each box in the figure represent the amount of attrition that occurred relative to the previous step in the study.

There were no differences in sociodemographic characteristics between the 2 treatment conditions in age, gender, ethnicity or minority status, education, or employment status. The baseline symptom profiles were also similar (Table 1). The average age of the participants was 44.11 years (SD 11.73) with a range of 22-77 years. Participants who identified as women were the largest gender represented, followed by men and then transgender individuals: 60.4% (122/202), 38.6% (78/202), and 0.5% (1/202), respectively. The majority of the participants 63.9% (129/202) had some college education or a high school diploma and 45.5% (92/202) were working full- or part-time. A total of 39.6% (80/202) of participants identified as a member of a racial or ethnic minority group. Treatment symptom measures did not differ between the 2 conditions with the exception of the WHODAS-21 results (Table 1). The participants who received Coach5 had higher WHODAS-21 scores (mean 49.38, SD 16.34) compared to those who received Coach10 (mean 44.97, SD 15.01; t_200_=2.00, *P*=.047). Baseline WHODAS-21 scores were included as a covariate in subsequent outcome models that compared treatment groups.

**Table 1:** Comparison of sociodemographic and baseline clinical characteristics of Coach5 and Coach10 participants.

Variable			Overall, N=202		Coach5, n=101		Coach10, n =101		*P* value (*t* test or chi-square test)	
Completed study, n (%)			100 (49.5)		54 (52.5)		47 (46.5)		.40	
Age, mean (SD)			44.10 (11.73)		44.96 (11.54)		43.06 (11.90)		.25	
**Gender, n (%)**										.08
	Male	78 (38.6)		33 (32.7)		45 (44.6)				
	Female	122 (60.4)		67 (66.3)		55 (54.5)				
	Transgender	1 (0.5)		0 (0)		1 (1)				
**Race, n (%)**										.12
	White	122 (60.4)		59 (58.4)		63 (62.4)				
	Black or African American	37 (18.3)		19 (18.8)		18 (17.8)				
	Asian	2 (1)		1 (1)		1 (2)				
	Hispanic, Latino(a)	11 (5.6)		6 (5.9)		5 (4.6)				
	Multiracial	17 (8.4)		13 (12.9)		4 (4)				
	Other^a^	13 (6.4)		3 (3)		10 (9.9)				
**Education level, n (%)**										.47
	Some high school	1 (0.5)		1 (1)		0 (0)				
	Earned high school degree	25 (12.4)		12 (11.9)		13 (12.9)				
	Some college/2-year degree	104 (51.5)		56 (55.6)		48 (47.5)				
	Earned 4-year degree	51 (25.3)		24 (23.8)		27 (26.7)				
	Postgraduate degree	21 (10.4)		8 (7.9)		13 (12.9)				
**Employment status, n (%)**										.26
	Full-time	73 (36.1)		31 (30.7)		42 (41.6)				
	Part-time	19 (9.4)		11 (10.9)		8 (7.9)				
	Not currently working	62 (30.7)		31 (30.7)		31 (30.7)				
	Retired	48 (23.8)		28 (27.7)		20 (19.8)				
**Relationship status, n (%)**										.12
	Married/partnered	115 (56.9)		52 (51.5)		62 (62.4)				
	Single	38 (18.8)		22 (21.8)		16 (15.8)				
	Divorced	47 (23.3)		25 (24.8)		22 (21.8)				
	Widowed	2 (1)		2 (2)		0 (0)				
**Baseline outcomes**										
	PCL^b^ total score, mean (SD) (range 2-78)	50.66 (15.52)		50.85 (16.09)		50.47 (15.01)		.86		
	PHQ^c^ total score, mean (SD) (range 2-27)	15.64 (5.40)		15.96 (5.38)		15.32 (5.44)		.40		
	DERS^d^ total score, mean (SD) (range 49-169)	108.74 (25.19)		108.72 (26.11)		108.75 (24.36)		.99		
	IIP-32^e^ score mean (SD) (range 0.22-3.31)	1.86 (0.53)		1.90 (0.53)		1.83 (0.53)		.38		
	WHODAS-2^f^ total score (SD) (range 0-81)	47.17 (15.81)		49.38 (16.34)		44.97 (15.01)		.05		

^a^Other included American Native Indian, Alaskan Native, Pacific Islander, Middle Eastern, and North African.

^b^PCL-5: posttraumatic stress disorder checklist for DSM-5

^c^PHQ-9: Patient Health Questionaire-9

^d^DERS: Difficulties in Emotion Regulation Scale

^e^IIP-32: Inventory in Interpersonal Problems-32

^f^WHODAS-21: brief version of World Health Organization Disability Assessment Schedule-2.

A total of 86.1% of participants (174/202) had a probable diagnosis of PTSD regardless of depression status and 86.6% (175/202) had a probable diagnosis of depression regardless of PTSD status. A total of 80.2% (162/202) had both disorders and 12.4% (25/202) had either one or the other, yielding a total of 92.6% (187/202) of the participants with at least one probable disorder. The study was completed by 49.5% (100/202) of participants. Compared to noncompleters, the completers were older (mean age 46.11 years vs 41.95 years; *P*=.01), more likely to rent versus own their home (97/100, 97% compared to 89/102, 87.3%; *P*=.01), more likely to be white (69/100, 69% vs 53/102, 52%; *P*=.01), and more likely to live in rural or highly rural locations (53/100, 67% vs 53/102, 52%; *P*=.03) Study completers and noncompleters were similar in all other demographic and pretreatment variables (*P*>.05 for all values, data not presented).

### Symptom Outcomes

Table 2 presents the observed mean for each outcome over time as well as within-group changes from baseline to posttreatment and from baseline to the 3-month follow up, organized by treatment group. All outcomes for both the Coach5 and Coach10 groups were significantly improved at the posttreatment assessment and the 3-month follow up relative to baseline. Furthermore, a series of ANCOVAs, controlling for baseline differences in WHODAS-21 scores, revealed no difference between the Coach5 and Coach10 groups in any of the 5 outcomes at the posttreatment assessment or at the 3-month follow up (see Table 3). ITT analyses revealed identical findings.

**Table 2:** Repeated measures tests and within-group effect sizes for the Coach5 and Coach10 groups.

	Outcome measure	Baseline: Coach5, n=101; Coach10, n=101	Posttreatment: Coach5, n=49; Coach10, n=46	Three-month follow up: Coach5, n=45; Coach 10, n=40	Within-group baseline to posttreatment		Within-group baseline to 3-month follow up	
					*P* value	Cohen *d* (95% CI)	*P* value	Cohen *d* (95% CI)
**PCL-5^a^score, mean (SD)**								
	Coach5	50.85 (16.09)	40.04 (19.50)	39.96 (19.84)	<.001	0.66 (0.38-0.94)	<.001	0.67 (0.37-0.97)
	Coach10	50.47 (15.01)	34.59 (19.66)	35.20 (19.42)	<.001	1.04 (0.73-1.35)	<.001	1.00 (0.67-10.33)
**PHQ-9^b^score, mean (SD)**								
	Coach5	15.96 (5.38)	12.39 (7.13)	12.58 (6.26)	<.001	0.65 (0.32-0.98)	<.001	0.62 (0.28-0.96)
	Coach10	15.32 (5.44)	9.91 (6.77)	10.35 (6.76)	<.001	0.65 (0.26-1.03)	0.003	0.90 (0.55-1.24)
**DERS-36^c^score, mean (SD)**								
	Coach5	108.72 (26.11)	90.51 (29.71)	88.13 (26.80)	<.001	0.69 (0.37-1.00)	<.001	0.69 (0.35-1.02)
	Coach10	108.75 (24.36)	88.07 (26.07)	85.85 (25.95)	<.001	0.83 (0.51-1.16)	<.001	0.92 (0.56-1.28)
**IIP-32^d^score, mean (SD)**								
	Coach5	1.90 (0.53)	1.67 (0.62)	1.58 (0.65)	.002	0.43 (0.15-0.70)	<.001	0.59 (0.33-0.85)
	Coach10	1.83 (0.53)	1.47 (0.65)	1.54 (0.65)	<.001	0.67 (0.36-0.72)	.001	0.54 (0.26-0.81)
**WHODAS-21^e^score, mean (SD)**								
	Coach5	49.38 (16.34)	44.14 (17.70)	42.46 (18.83)	.001	0.32 (0.06-0.57)	<.001	0.42 (0.17-0.66)
	Coach10	44.97 (15.01)	35.43 (19.33)	35.03 (18.62)	.04	0.62 (0.30-0.95)	.04	0.65 (0.35-0.95)

^a^PCL-5: posttraumatic stress disorder checklist for DSM-5

^b^PHQ-9: Patient Health Questionaire-9

^c^DERS: Difficulties in Emotion Regulation Scale

^d^IIP-32: Inventory in Interpersonal Problems-32

^e^WHODAS-21: brief version of World Health Organization Disability Assessment Schedule-2

**Table 3:** Between-group effect sizes at posttreatment assessment and 3-month follow up.

Outcome measure	Coach 5 vs Coach 10 at posttreatment assessment			Coach 5 vs Coach 10 at 3-month assessment		
	*F* test (*df*)	Treatment group *P* value	Cohen *d* (adjusted)	*F* test (*df*)	Treatment group *P* value	Cohen *d* (adjusted)
PCL-5^a^ total score	0.06 (1,91)	.81	0.05	0.01 (1,81)	.93	0.02
PHQ-9^b^ total score	0.07 (1,91)	.80	0.05	0.05 (1,81)	.82	0.05
DERS-36^c^ total score	0.05 (1,91)	.83	0.05	0.69 (1,81)	.41	0.18
IIP-32^d^ mean score	0.03 (1,91)	.87	0.03	3.00 (1,81)	.09	0.38
WHODAS-21^e^ total score	0.27 (1,92)	.60	0.11	0.31 (1,82)	.58	0.12

^a^PCL-5: posttraumatic stress disorder checklist for DSM-5

^b^PHQ-9: Patient Health Questionaire-9

^c^DERS: Difficulties in Emotion Regulation Scale

^d^IIP-32: Inventory in Interpersonal Problems-32

^e^WHODAS-21: brief version of World Health Organization Disability Assessment Schedule-2

### Tests of Noninferiority

**Figure 2:**
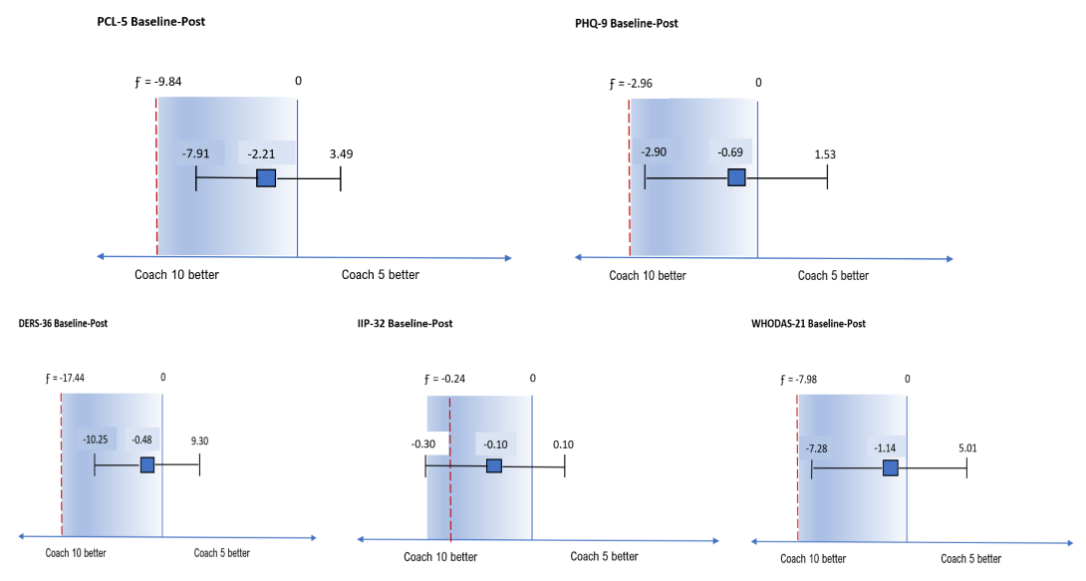
Noninferiority figures for 5 outcomes. PCL-5: posttraumatic stress disorder checklist for DSM-5; PHQ-9: Patient Health Questionaire-9; DERS: Difficulties in Emotion Regulation Scale; IIP-32: Inventory in Interpersonal Problems-32; WHODAS-21: brief version of World Health Organization Disability Assessment Schedule-2.

Figure 2 shows the CIs for the mean difference in scores for the 5 outcome measures between conditions for the completer sample at posttreatment assessment. If Coach10 is superior to Coach5, the difference in the change score is negative. The value of *F* (shown as the dotted vertical line) is the predetermined minimum clinically significant difference (ie, the margin of noninferiority). For 4 of 5 outcomes, the lower bound of the 95% CI was less than 0 and yet also greater than the margin of noninferiority (ie, it is in the shaded area) and the higher bound of the 95% CI was greater than 0, which indicates noninferiority of Coach5 with respect to Coach10 in improvement from baseline to posttreatment assessment. For the IIP, the lower bound of the 95% CI was less than 0 but also less than the margin of noninferiority (ie, it crossed the margin of inferiority), which indicates inconclusive results.

When examining changes from baseline to the 3-month follow up, the lower bound of the 95% CI was within the shaded area for PCL-5, DERS-36, IIP-32, and WHODAS-21, indicating the noninferiority of Coach5 with respect to Coach10 in improvement from baseline to the 3-month follow up for these 4 outcomes. Conversely, for the PHQ-2 the lower bound of the 95% CI was less than the margin of noninferiority (ie, it crossed the margin of noninferiority), which indicates inconclusive results.

### Therapeutic Alliance

The average WAI score in the Coach5 and Coach10 conditions was not significantly different, with a mean of 6.66 (SD 0.49) and 6.54 (SD 0.50) respectively (t_128_=1.34, *P*=.18). The score for WAI items ranged from 1 to 7, with a higher score indicating a better relationship. The results of this assessment indicate that participants’ perception of the working alliance with their therapists was very high, and equally so, across the 2 conditions.

### Completion Rates

The total number of modules completed by the Coach5 and Coach10 groups did not differ (6.88, SD 4.19 vs 6.66, SD 4.11, respectively; t_200_=0.37, *P*=.71). The percentage of participants who completed half of the program (up to and including the fifth module) did not differ between the Coach5 (72/101, 71.3%) and Coach10 (67/101, 66.4%) groups. The total percentage of participants who completed all 10 modules also did not differ between the Coach5 (59/101, 58.4%) and Coach10 (52/101, 51.5%) groups (*χ*^2^_1_=0.93, *P*=.32).

### Web Usage

Coach5 participants spent an average of 31.07 minutes (SD 8.30) per module with time per module ranging from 43.66 to 18.80 minutes. Coach10 participants spent an average of 33.06 minutes (SD 11.05) per module with a range of 38.98 to 14.36 minutes. The average amount of time participants who completed the program spent on the website was 341.83 minutes (SD 160.46) for Coach5 and 363.63 (SD 160.46) for Coach10. The difference in total time spent on the program between the 2 conditions was not significant (t_20_ =2.02, *P*=.16).

### Therapist Session Time and Activities

As expected, the Coach5 therapists spent significantly less time than the Coach10 therapists in session time (t_28_=28.44, *P*<.001), with the Coach5 therapists spending an average of 332.67 minutes (SD 49.10) across the 5 coaching sessions and the Coach 10 therapists spending an average of 505.09 minutes (SD 111.79) across all 10 sessions. The number of contacts (phone calls, instant messages, or emails) did not differ between the Coach5 and the Coach10 therapists, with values of 2.83 (SD 3.28) and 2.02 (SD 2.85), respectively. The number of phone calls for interventional purposes (crisis management or motivational interventions) was low and did not differ between the Coach5 (M 1.10, SD 1.65) and Coach10 therapists (M 0.62, SD 1.06).

### Therapist Adherence Ratings

The adherence rating indicates the percentage of intervention items that were completed by the therapist in each session. Overall, the average adherence ratings were high and were statistically similar across both the Coach5 (M 0.90, SD 0.14) and Coach10 conditions (M 0.97, SD 0.06).

## Discussion

Five sessions of therapist support for a transdiagnostic trauma-informed intervention, delivered to veterans with moderate to severe symptoms of PTSD, depression, or both was found to be noninferior to a 10-session delivery approach across several outcomes. Participants obtained significant benefits from webSTAIR with both conditions. In addition, therapeutic alliance was strong and did not differ between 5-session and 10-session delivery. The time participants spent on the program was equivalent, as was the completion rate. This study demonstrates the feasibility and effectiveness of blended models in routine clinical care for patients with moderate to severe symptoms. It also contributes to the exploration of the therapist role in technology-based intervention and provides parameters regarding the amount of therapist support that is associated with good outcomes for patients.

Symptom reduction was significant for PTSD, depression, emotion regulation, interpersonal problems, and overall functioning at both posttreatment and a 3-month follow up for both treatment conditions and was associated with predominantly moderate to large effect sizes. Most of the measures demonstrated noninferiority at both posttreatment and follow up. Two measures, interpersonal problems and depression, were associated with some variability, indicating either noninferiority or an inconclusive result depending on the timepoint. However, overall, all 5 outcomes for the Coach5 group were not inferior to the Coach 10 group at any time point.

On average, participants spent approximately 30 minutes per module and completed about 6 to 7 modules. The percentage of participants who completed the entire program (ie, all 10 modules) was 58% (59/101) for the Coach5 group and 51% (51/101) for the Coach10 group. The level of success that this represents is unknown. Because the project was an evaluation program, not a research study, there were no special efforts made to retain participants in the project (eg, ensure before enrollment that a participant had no plans for long travel or hospitalization that might disrupt completion), as would occur in a research study. RCTs of internet-based interventions for PTSD populations have reported drop-out rates of 25%, and by this measure, the completion rates in this study are inferior. On the other hand, in studies of naturalistic use of technology-based interventions, retention rates appear to fall to 20% by the fifth session [35], and by this measure, our completion rate is superior. Additional studies are needed to determine the completion rates associated with blended therapies delivered in a clinical service context.

It is notable that the large majority of patients enrolled in the program reported severe symptoms of PTSD, depression, or both, with the overwhelming majority (187/202, 92.6%) meeting criteria for a probable diagnosis of one or the other disorder and over 80% (162/202) meeting a probable diagnosis for both. The results indicate that either of these blended approaches is effective and safe for highly symptomatic patients. It is often thought that technology-based therapies are most appropriate primarily for individuals with low to moderate symptoms. Technology-based programs have typically been introduced into mental health services as part of “stepped care” delivered to enrolling patients whose symptoms are relatively mild or as part of maintenance care once face-to-face treatment has been completed. Our findings indicate that technology-based care with therapist support is effective even for severely affected patients. It is interesting to speculate whether referral of relatively severe patients to this program by clinical therapists was motivated by knowledge of high level of therapist involvement. The blended therapy model may introduce a new way of thinking about mental health care that provides high quality care to patients but reduces therapist burden in terms of time and effort.

The overall noninferiority of Coach5 compared to Coach10 provides therapists and mental health services reassurance that a reduction in therapist time does not lead to a reduction in good outcomes for patients or in the relationship with the patient. Therapists have been concerned that reduction in therapist time might lead to increased need to manage crisis events [19,20], representing a reduction in quality of care for patients and their well-being as well as a “hidden cost” in regard to therapist time and effort. However, the Coach5 and Coach10 programs were associated with a relatively low need for additional support and intervention, with therapists in both conditions reporting on average 1 phone session per patient and 3 contacts related to rescheduling of sessions in addition to the technology-based program.

Finally, the finding of noninferiority may be viewed as counterintuitive if one assumes a “dose-response” effect in psychotherapy treatment, where more therapist intervention is better. We anchored the study to a 1:1 ratio of therapist session to self-guided work. This ratio represents the maximum dose of therapist support in a program in which the technology intervention is primary. Its selection was supported by evidence from previous studies indicating the success of this ratio [15,36]. The absence of worse outcomes with a reduction in the number of therapist sessions can be interpreted in several ways. However, one possibility is that there are mechanisms at work other than an additive dose-response effect. For example, it is possible that self-guided work and the therapist sessions made unique and complementary contributions to the program outcomes and may ultimately have provided greater benefits than therapist-only or internet-only approaches [18]. Evidence for this view is supported by qualitative analyses from interviews completed in one of our earlier studies [21], which found that the self-guided work facilitated a sense of autonomy and mastery, while the therapist sessions provided emotional and practical support as well as clarity in tailoring the tools to specific problems and life experience. The potential presence of a dynamic, reciprocal relationship between these 2 treatment components deserves further investigation via assessment of cross-lagged effects and identification of underlying mechanisms of change.

The strengths of this study include the delivery of the treatment in a usual care context, provided by clinic staff and delivered to trauma-exposed patients with relatively severe and diverse symptoms and problems. Two limitations of the study are the absence of randomization, which could have resulted in unidentified patient factors influencing outcomes, and the potential lack of generalizability of the findings from veterans to other trauma-exposed populations. Future studies are warranted evaluating other combinations of therapist-supported and self-guided work and delivery to different trauma populations. In addition, studies identifying patient characteristics as moderators of outcomes in high versus low doses of therapist support will help create flexible technology-based intervention programming that facilitates engagement of a greater number of individuals and tailoring of therapist time and attention relative to client need.

## Abbreviations

CBT: cognitive behavioral therapy
DERS: Difficulties in Emotion Regulation Scale
DSM-5: Diagnostic and Statistical Manual, Fifth Edition
IIP: Inventory of Interpersonal Problems
ITT: intent-to-treat
PCL-5: PTSD Checklist for DSM-5
PHQ: Patient Health Questionnaire
PST: problem-solving therapy
PTSD: posttraumatic stress disorder
RCT: randomized controlled trials
WAI: Working Alliance Inventory
WHODAS: World Health Organisation Disability Assessment Schedule
